# Noninvasive Tests (NITs) for Hepatic Fibrosis in Fatty Liver Syndrome

**DOI:** 10.3390/life10090198

**Published:** 2020-09-13

**Authors:** Ma Ai Thanda Han

**Affiliations:** 1 Division of Gastroenterology and Hepatology, Rutgers New Jersey Medical School, 185 South Orange Avenue, H-532, Newark, NJ 07103, USA; maaithandahan@gmail.com; Tel.: +1-917-355-1122; 2 Division of Gastroenterology and Hepatology, Mayo Clinic, Rochester, MN 55905, USA

**Keywords:** fatty liver syndrome, non-alcoholic fatty liver disease, alcoholic liver disease, hepatic fibrosis, hepatic steatosis

## Abstract

Fatty liver syndrome is an emerging health problem in the world, due to the high prevalence of obesity and alcohol use disorder. Given the nature of the disease’s advancement to cirrhosis and liver-related complications, it is important to assess the severity of the disease, which is typically done via a liver biopsy. Due to the limitations and risks of liver biopsy, the role of noninvasive tests is essential and evolving to stratify the stage of the liver disease, predict the outcomes, and/or monitor the treatment response. This review is focused on noninvasive tests, including the use of serum-based biomarkers, ultrasound-based shear wave elastography, transient elastography, and magnetic resonance elastography in both clinical and research settings.

## 1. Introduction

Hepatic steatosis is found in both non-alcoholic fatty liver (NAFLD) and alcoholic liver disease (ALD), both of which can coexist. The manifestations of both non-alcoholic fatty liver (NAFLD) and alcoholic liver disease (ALD) are the same, including simple steatosis to steatohepatitis with or without fibrosis, cirrhosis, and hepatocellular carcinoma. Therefore, we use the term “Fatty Liver Syndrome” to cover both NAFLD and ALD. The health burden of fatty liver syndrome is increasing globally along with the emerging prevalence of obesity and alcohol use disorder. Fatty liver syndrome is becoming one of the most common etiologies of chronic liver disease and liver transplantation [1,2,3,4]. Given the natural history of the disease, which can progress to an advanced liver disease and develop complications, it is essential to assess the severity of the disease. The degree of hepatic fibrosis, regardless of other histologic features (such as steatohepatitis), is the most important variable to stratify the risk, as this factor can predict the mortality and long-term outcomes of patients with fatty liver syndrome [5,6,7,8]. 

Liver biopsy is the gold standard to evaluate hepatic fibrosis. Since it is an invasive procedure and has limitations, including a risk of complications, sampling errors, low acceptance by patients, and inconvenience [9,10], non-invasive tests have been investigated to stratify the stage of hepatic fibrosis. The histologic staging of hepatic fibrosis has been used in phase 3 clinical trials, but magnetic resonance elastography (MRE)-based staging of hepatic fibrosis is used currently in phase 2 clinical trials [2,11]. In this article, we review non-invasive fibrosis tests (NITs), which are mainly categorized into tests of serum-based biomarkers and imaging tests. Serum-based biomarkers include both simple and complex serum-based biomarkers. The imaging tests include shear wave elastography (SWE), transient elastography (TE), and magnetic resonance elastography (MRE). 

## 2. Serum Based Biomarkers

### 2.1. Simple NIT Biomarkers

Simple non-invasive biomarkers are based on patients’ demographics and simple routine blood tests. Patient demographics include age, sex, body mass index (BMI), history of diabetes mellitus, and simple routine blood tests, including alanine aminotransferase (ALT), aspartate aminotransferase (AST), platelet counts, fasting glycemia, and albumin. The NAFLD fibrosis score (NFS), AST to platelet ratio index (APRI), FIB4-index, BARD score, and AST/ALT ratio (AAR) were explored among the patients with NAFLD. The APRI, FIB4-index, and Forns index were explored among patients with ALD. A summary of the accuracy is shown in Table 1. 

#### 2.1.1. NAFLD Fibrosis Score (NFS)

The NAFLD fibrosis score (NFS) is based on age, BMI, impaired fasting glycemia or diabetes mellitus, AST/ALT ratio, platelet count, and the serum level of albumin. NFS is calculated as 1.675 + 0.0373 × age (years) + 0.0943 BMI (kg/m^2^) + 1.133 × impaired fasting glycaemia or diabetes (yes = 1, no = 0) + 0.993 × AST/ALT ratio − 0.0133 × platelet (×10^9^/L) − 0.663 × albumin (g/dL) [12]. 

In a large multi-center international cohort with 733 biopsy-proven NAFLD patients, there were two cut-offs identified to predict the severity of hepatic fibrosis. The low cut-off score (<−1.455) projected no/mild fibrosis (F0–F2) with a 93% and 88% negative predictive value (NPV) in the estimation and validation cohorts, respectively. The high cut-off score (>0.676) projected advanced fibrosis (F3–F4) with a 90% and 82% positive predictive value (PPV) in the estimation and validation cohorts, respectively. This score provided an AUROC of 0.84 and the potential to avoid liver biopsy in 75% of patients [12]. A more recent study of 145 biopsy-proven NAFLD patients also demonstrated 92% NPV and 72% PPV, with an AUROC of 0.81. The AUROC of NFS was better than that of the BARD score (0.77) and that of APRI (0.67) [13]. Similarly, a high predictive value with good accuracy was found in studies on Asian populations [14].

#### 2.1.2. AST to Platelet Ratio Index (APRI)

The AST to Platelet Ratio Index (APRI) score is generated to measure hepatic fibrosis in chronic hepatitis C with cut-offs of 1.5 to predict significant fibrosis and 2.0 to predict cirrhosis. The APRI is calculated as ((AST/upper limit normal AST) × 100)/Platelet (10^9^/L) [15]. The score was adopted for both the NAFLD and ALD populations.

The performance of APRI among the 235 NAFLD patients demonstrated AUROCs of 0.866, 0.861, and 0.842 in predicting significant fibrosis, severe fibrosis, and cirrhosis respectively. In total, 80% of patients had the potential to avoid liver biopsy [16]. However, other studies revealed lower performance, with AUROCs of 0.564, 0.568, and 0.786 for significant fibrosis, advanced fibrosis, and cirrhosis, respectively [17,18]. A recent study by McPherson et al., exhibited AUROC of 0.67, which is lower than the AUROCs of FIB4, NFS, and BARD score used to predict hepatic fibrosis ≥F3 [13]. 

The sensitivity and specificity of the APRI score in ALD were low, with 35.6% and 29.7%, respectively, which is most likely due to frequent alcohol use. The AUROC was also low, with 0.59 for detecting significant fibrosis (F ≥ 2) and 0.67 for detecting cirrhosis [19,20]. However, a study of 289 patients with ALD by Thiele et al., demonstrated an AUROC of 0.8 for detecting advanced fibrosis (F ≥ 3) and 0.85 for detecting cirrhosis. Most of the patients in this study had mild liver disease, which is the main limitation of this study [21].

#### 2.1.3. FIB4-Index

The FIB4-index is composed of age along with the serum markers of platelets, ALT, and AST. The FIB4-index is calculated as Age × AST (IU/L)/platelet count (×10^9^/L) × √ALT (IU/L) [22].

In the nationwide data of 541 NAFLD patients, a low cut-off of 1.3 excludes advanced fibrosis with 74% sensitivity, 71% specificity, and 90% NPV. The high cut-off of 2.67 to include advanced fibrosis demonstrated 33% sensitivity, 98% specificity, and 80% PPV. The AUROC was 0.802 [22]. A recent study by McPherson et al. also presented an AUROC of 0.86 for including advanced fibrosis (F ≥ 3). This index was able to exclude advanced fibrosis in 62% of patients [13]. 

In 218 patients with ALD, the FIB4-index provided AUROCs of 0.7 and 0.8 for distinguishing significant fibrosis (F ≥ 2) and cirrhosis (F4), respectively, which is higher than the values of the APRI and Forns index [20]. A similar result was shown by Thiele et al., with AUROCs of 0.85 and 0.89 identified to detect advanced fibrosis (F ≥ 3) and cirrhosis, respectively [21].

#### 2.1.4. BARD Score

A combination of BMI, the AST/ALT ratio, and a history of diabetes mellitus are designed into the BARD score to identify hepatic fibrosis. The BARD is derived from the sum of three variables (BMI > 28 = 1 point, AST/ALT ratio > 0.8 = 42 points, diabetes = 1 point) [23].

A multicenter study of 827 patients with NAFLD showed an association between a BARD score of 2–4 and advanced fibrosis (F ≥ 3) with an OR of 17, 96% NPV, and an AUROC of 0.81, but a low PPV of 43% [23]. The advantage of this score is the lack of an indeterminate zone as there are no two cut-offs. This score was applied to the Japanese population with NAFLD. The OR was 4.9, and it provided an AUROC of 0.73 with 59% PPV and 77% NPV [24]. In comparison with other scores, the AUROC of the BARD score (0.77) was better than that of APRI (0.67), but that of the BARD score was lower than those of the FIB4-index (0.86) and NFS (0.81) used to predict advanced fibrosis [13]. 

#### 2.1.5. AST/ALT Ratio (AAR)

The AST/ALT ratio (AAR) is one of the simplest methods to assess hepatic fibrosis using a widely available blood test. 

Two cut-offs of 0.8 or 1 are used to exclude or include advanced fibrosis among patients with NAFLD. Initially, the AUROC was found to be 0.742 by Shah et al. [22]. In another study comparing the AAR with different other simple scores, the AUROC was 0.83 with 93% NPV [13]. Based on data from NASH research network (NASH CRN) multi-center studies, the AUROCs were 0.73 and 0.81 to predict advanced fibrosis and cirrhosis, respectively, while using a cut-off of 1 [25]. Among the patients with ALD, the AUROC to predict advanced fibrosis (F ≥ 3) and cirrhosis (F4) was 0.76 [21].

#### 2.1.6. Forns Index

The Forns index includes four clinical parameters: age, platelet count, total cholesterol, and gamma glutamyl transferase (GGT). The Forns index is calculated as 7.811 − 3.131 × ln (platelet count) + 0.781 × ln (GGT) + 3.467 × ln (age) − 0.014 × (total cholesterol) [26].

In a study of 218 patients with ALD and a median follow up 8.2 years, the performance was poor, with an AUROC of 0.38 [20]. However, in another recent study that included 289 patients from both primary and secondary care centers, most of whom had mild disease activity or injury, the AUROCs were 0.85 and 0.89 to identify advanced fibrosis (F ≥ 3) and cirrhosis (F4), respectively [21].

Among the simple NIT biomarkers, the AUROCs of FIB-4 and NFS are the highest for NAFLD patients and the AUROC of the FIB-4 index is the highest for ALD patients. 

### 2.2. Complex NIT Biomarkers

Complex non-invasive biomarkers include FibroTest/FibroSure, fibrospect, enhanced liver fibrosis panel (ELF), pro-C3 based predictive fibrosis score, and NIS4 for patients with NAFLD and the PGA index, PGAA index, FirboTest/FibroSure, and ELF for patients with ALD. A summary of the accuracy is shown in Table 2.

#### 2.2.1. FibroTest/FibroSure

FibroTest is designed using a combination of total bilirubin, gamma glutamyl transferase (GGT), alpha-2macroglobulin, apolipoprotein A1, and haptoglobin, adjusted for age and gender. 

The performance of the FibroTest was studied in a small sample of 97 biopsy-proven NAFLD patients. The AUROC was 0.81 to identify advanced fibrosis (F ≥ 3). The FibroTest cut-off of 0.3 provided a 90% NPV, and a cut-off of 0.7 provided a 73% PPV to identify advanced fibrosis (F ≥ 3) [27].

The performance in the 218 patients with ALD also provided an AUROC of 0.83 to identify advanced fibrosis and an AUROC of 0.94 to identify cirrhosis, which indicates significantly better performance than that of the APRI, Forns, and FIB4-index (*p* < 0.01) [20]. Another recent study also showed a similar result, with an AUROC of 0.88 to identify advanced fibrosis and cirrhosis [21].

#### 2.2.2. Fibrospect

Fibrospect is based on an algorithm encompassing the concentrations of three serum markers, alpha-2 macroglobulin (A2M), hyaluronic acid (HA), and tissue inhibitor metallopeptidase protein 1 (TIMP1), to differentiate NAFLD patients with advanced fibrosis from those with milder forms of fibrosis. In a retrospective analysis of 396 patients with NAFLD, the AUROC of the validation cohorts was 0.867, with 79.7% sensitivity and 75.7% specificity and 92.5% to 94.7% NPV, with a cutoff score of 17. Compared to the AUROCs for simple NIT biomarkers, that of Fibrospect (0.85) is better than those of the FIB4-index (0.77) and NFS (0.61) [28].

#### 2.2.3. Enhanced Liver Fibrosis Panel (ELF)

The enhanced liver fibrosis panel was developed from an algorithm involving three serum markers (hyaluronic acid (HA), amino-terminal peptide pro-collagen 3 (P3NP), and tissue inhibitor metallopeptidase protein 1 (TIMP1)) to distinguish advanced fibrosis from early stages of fibrosis.

Initial data from the European liver fibrosis group on NAFLD patients showed two different cut-offs. A score below the cut-off of 0.375 suggested no/mild fibrosis with 89% sensitivity, 96% specificity, 80% PPV, and 98% NPV. A score above the cut-off of 0.462 suggested moderate/severe fibrosis with 78% sensitivity, 98% specificity, 87% PPV, and 96% NPV. This study achieved an AUROC of 0.87 [29]. Recently, the paired serum and histological data of 192 patients with NAFLD were explored in medical centers from the United Kingdom. The AUROC to detect advanced fibrosis was 0.9 with 80% sensitivity, 90% specificity, 71% PPV, and 94% NPV, respectively, at a cut-off 0.3576. The AUROC was 0.82 with a PPV of 70% to detect moderate fibrosis at a cut-off of −0.1068, and the AUROC was 0.76 with a PPV of 81% to detect patients without fibrosis [30]. 

The European liver fibrosis group also demonstrated the good performance of ELF on patients with ALD. A score below the cut-off of 0.087 suggested no/mild fibrosis with 100% sensitivity, 16.7% specificity, 75% PPV, and 100% NPV. A score above the cut-off of 0.431 suggested moderate/severe fibrosis with 93.3% sensitivity, 100% specificity, 100% PPV, and 85.7% NPV [29]. The accuracy of ELF among 289 patients with ALD was again confirmed recently by Thiele et al. The AUROC to identify advanced fibrosis was 0.92, and that to identify cirrhosis was 0.94. When the AUROC of ELF was compared with the AUROCs of the other serum NIT biomarkers, including the AST/ALT ratio (0.76), APRI (0.8), FIB-4 index (0.85), Forns index (0.86), and FibroTest (0.88), ELF was best able to identify advanced fibrosis (F ≥ 3) and cirrhosis [21].

#### 2.2.4. PRO-C3 Based Predictive Fibrosis Score

PRO-C3 is a marker of type III collagen formation and is independently associated with advanced fibrosis in NAFLD. An algorithm was generated based on age, history of diabetes mellitus, PRO-C3, and platelet count (ADAPT) to distinguish patients with advanced fibrosis from patients with no or mild fibrosis. Recently, 431 biopsy-proven NAFLD patients were studied for their PRO-C3 based predictive fibrosis scores. Patients with a high PRO-C3 possessed a 1.5-fold higher chance of having advanced fibrosis. The PRO-C3 based predictive fibrosis score (ADAPT) achieved an AUROC of 0.86 and 0.87 to detect advanced fibrosis in the derivation cohort and validation cohort, respectively. Compared to simple NIT biomarkers, such as the FIB-4 index, NFS, and APRI, the performance of ADAPT was superior, providing an AUROC of 0.86 compared to the that of the FIB-4 index (AUROC 0.73), NFS (AUROC 0.78), and APRI (AUROC 0.78) [31].

#### 2.2.5. NIS4^TM^

A new score, NIS4^TM^, uses the biomarkers of miR-34a, alpha-2 macroglobulin, YKL40, and HbA1C to detect active steatohepatitis and advanced fibrosis (NAFLD activity score ≥4 and fibrosis ≥2). MicroRNA-34a (miR-34a) represents a marker for steatosis, inflammation, and hepatocyte ballooning; alpha-2 macroglobulin and YKL40 represent markers for hepatic fibrosis, and YKL40 is also involved in inflammation and tissue remodeling in response to endothelial dysfunction. Moreover, HbA1C represents a marker for insulin resistance. An algorithm was generated based on these markers to efficiently differentiate between advanced disease and mild disease. The data were generated from two prospective trials—GOLDEN-505 and RESOLVE-IT—comprising 714 NAFLD patients. The AUROC for detecting active steatohepatitis and advanced fibrosis was 0.826. Below the low cut-off of 0.3642, 60% of the patients did not have active steatohepatitis or advanced fibrosis, with an NPV of 79.5%. Above the high cut-off of 0.6137, 60% of patients had active disease, with advanced fibrosis having a PPV of 81.2%. Finally, a head-to-head comparison of NIS4^TM^ with other pre-existing NIT scores, including the FIB4-index, NFS, ELF, BARD, and APRI, showed that NIS4^TM^ statistically significantly outperforms the rest of the scores in identifying active steatohepatitis with advanced fibrosis (*p* < 0.001) [32].

#### 2.2.6. PGA Index and PGAA Index

The PGA index includes prothrombin time, GGT, and Apolipoprotein A [33]. The addition of alpha 2 macroglobulins to the PGA index was done to obtain PGAA index, which provided better performance in detecting hepatic fibrosis than the PGA index in patients with ALD. The AUROCs of the PGAA index were 0.83 and 0.87 for identifying significant fibrosis (F ≥ 2) and cirrhosis (F4), respectively. Compared to FibroTest, there was no statistically significant difference in the results [34].

The study of these complex NIT biomarkers is still emerging. A head to head comparison in the performance of these complex NIT biomarkers has not been studied yet. Non-invasive serum biomarkers essentially generate two different cut-offs to predict either no/mild hepatic fibrosis (F0–F2) or advanced fibrosis (F3–F4). The main challenge for these simple fibrosis markers is the difficulty interpreting the indeterminate zone, which is the score that lies between the two cut-offs. A liver biopsy is recommended for patients whose score falls within the indeterminate zone. Therefore, a sequential non-invasive test was explored to reduce the indeterminate zone. Data analyzed from two clinical trials (STELLAR-3 and STELLAR-4) found that single non-invasive tests (NFS, FIB4-index, ELF, or VTE) provide an indeterminate zone in up to 50% of patients. A sequential non-invasive test using FIB-4 followed by ELF reduced the indeterminate zone to 24%, and FIB4 followed by transient elastography reduced the indeterminate zone to 20% [35]. Further well-designed studies are thus warranted. 

## 3. Imaging Tests

Conventional non-invasive imaging tests including conventional ultrasound and computed tomography (CT) of abdomen are unable to detect early stage of liver fibrosis. They can detect only late stage of hepatic fibrosis (cirrhosis) and/or complications such as portal hypertension [36]. CT perfusion image is a functional imaging test able to detect an alteration of hepatic microcirculation in different stages of fibrosis. The image was studied in patients with chronic viral hepatitis B and C, suggesting promising accuracy [37,38]. Another study also showed that the splenic CT perfusion parameters correlated well with hepatic venous pressure gradient (HVPG) and was able to detect severe portal hypertension, which is HVPG ≥ 12 mmHg in 21 cirrhotic patients with mixed etiologies of liver disease [39]. However, there is no well-designed study of CT perfusion image to identify different stages of hepatic fibrosis and/or portal hypertension in patients with either non-alcoholic or alcoholic fatty liver disease. Therefore, this review article is essentially focused on the non-invasive imaging tests studied in patients with fatty liver syndrome, including shear wave elastography (SWE), transient elastography (TE), and magnetic resonance elastography (MRE). 

### 3.1. Shear Wave Elastography (SWE)

Shear wave elastography (SWE) is an ultrasound-based elastographic method that uses acoustic radiation force impulse (ARFI) techniques. These techniques include point shear wave elastography (pSWE) and two-dimensional (2D) shear wave elastography (SWE) at frequencies of 100–500 Hz. The sampling area of the liver in pSWE is small compared to that using 2D SWE. The ultrasound can flexibly locate the area of interest, which is 1 cm below the liver capsule and <5 cm from the transducer. pSWE measures the area of attenuation and the shear wave velocity in meter/second (m/s), whereas 2D SWE reports liver stiffness using the Young’s modulus in kilopascals [40,41].

The accuracy of pSWE was initially measured in 54 biopsy-proven NAFLD patients, showing an AUROC of 0.973 for identifying advanced fibrosis (F ≥ 3) at an optimal cut-off of 1.77 m/s with 100% sensitivity and 91% specificity. The AUROC became 0.976 when identifying cirrhosis at an optimal cut-off of 1.90 m/s with 100% sensitivity and 96% specificity [42]. In another study comparing pSWE with transient elastography (TE), there was no statistically significant difference in accuracy between the two; however, the correlation of liver stiffness with histologic fibrosis was better in TE than in pSWE [43]. In a meta-analysis of three studies that used SWE and AUROCs to determine significant fibrosis (F ≥ 2), advanced fibrosis (F ≥ 3) and cirrhosis (F4) were 0.89, 0.91, and 0.97, respectively, which are higher scores than those determined using transient elastography (with an M or XL probe). The sensitivity, specificity, PPV, and NPV were 85–100%, 85.6–94.4%, 55.2–93.9%, and 84.8–100%, respectively. However, the cut-offs to identify significant fibrosis (F ≥ 2) (2.67–9.4) and advanced fibrosis (≥F3) (5.7–9.3) widely varied among the three studies [44]. The data on two-dimensional SWE remain limited. There are only a few studies on 2D SWE provided an AUC of 0.89 with 100% sensitivity, 85% specificity, 55.2% PPV, and 100% NPV [40,45].

In patients with ALD, the data are also limited and not robust. Among 112 patients with biopsy-proven ALD, the AUROCs of pSWE were 0.84, 0.87, and 0.89 for identifying significant fibrosis (F ≥ 2), advanced fibrosis (F ≥ 3), and cirrhosis (F4), respectively. However, this study revealed that elevated ALT affects the results of pSWE [46]. In a prospective study with 119 ALD patients using 2D SWE, the cut-offs to identify significant fibrosis (F ≥ 2) and cirrhosis (F4) were 10.2 kPa and 16.4 kPa, respectively, with AUROCs of more than 0.92. The accuracy of 2D SWE was compared with that of transient elastography, showing no statistically significant difference between them [47].

### 3.2. Transient Elastography (TE)

Transient Elastography (TE) or Fibroscan is another ultrasound-based method used to measure liver stiffness. The shear wave propagates through the liver parenchyma with transducer-induced vibrations at a low frequency of 50 Hz and low amplitudes. TE measures the liver parenchyma in an area 1 cm wide and 4 cm long. A reliable value is obtained from ≥10 valid measurements with a success rate of >60%. TE provides the value of liver stiffness in kilopascals (kPa). The major limitations of TE include obesity, significant ascites, and the results being influenced by significant inflammation or congestion along with a post-prandial state [11,48,49]. 

In a meta-analysis of seven studies using TE in NAFLD patients, a cut-off of 6.7–7.7 kPa can identify significant fibrosis (F ≥ 2) with 79% sensitivity and 75% specificity; a cut-off of 8–10.4 kPa can identify advanced fibrosis (F ≥ 3) with 85% sensitivity and 82% specificity; and, finally, a cut-off of 10.3–17.5kPa can identify cirrhosis (F4) with 92% sensitivity and 92% specificity [50]. Another study conducted a meta-analysis of two separate TE results with an M probe and XL probe. When using the M probe, the cut-offs for significant fibrosis (F ≥ 2) were 5.8–11 kPa with an AUROC of 0.82, the cut-offs for advanced fibrosis (F ≥ 3) were 6.95–11.4 kPa with an AUROC of 0.87, and the cut-offs for cirrhosis were 7.9–22.3 kPa, with an AUROC of 0.92. When the XL probe was used, the cut-offs for significant fibrosis (F ≥ 2) were 4.8–8.2 kPa with an AUROC of 0.82, the cut-offs for advanced fibrosis (F ≥ 3) were 5.7–9.3 kPa with an AUROC of 0.86, and finally, the cut-off for cirrhosis was 7.2–16 kPa, with an AUROC of 0.94 [44]. A more recent multi-center study of 415 patients with NAFLD explored the sensitivity threshold and Youden’s threshold using appropriate M or XL probes to score the optimal cut-off for a better stratification of hepatic fibrosis. A sensitivity threshold of 6.1 kPa was used to rule out significant fibrosis (F ≥ 2) (90% sensitivity, 38% specificity, 0.67% PPV, and 0.72% NPV), 7.1 kPa to identify advanced fibrosis (F ≥ 3) (90% sensitivity, 75% specificity, 63% PPV, and 81% NPV), and 10.9 kPa to identify cirrhosis (F4) (91% sensitivity, 70% specificity, 23% PPV, and 99% NPV). When using Youden’s threshold, a cut-off of 8.2 kPa was identified to rule out significant fibrosis (F ≥ 2) with an AUROC of 0.77, 71% sensitivity, 70% specificity, 78% PPV, and 61% NPV. Advanced fibrosis (F ≥ 3) was identified at a cut-off of 9.7 with an AUROC of 0.8, 71% sensitivity, 75% specificity, 63% PPV, and 81% NPV. Finally, cirrhosis (F4) was identified under a cut-off of 13.6 kPa with an AUROC of 0.89, 85% sensitivity, 79% specificity, 29% PPV, and 98% NPV [51]. Recently, a combination AST and fibroscan, the FibroScan–AST (FAST) score, was developed to identify active disease defined by steatohepatitis (NASH), an elevated NAFLD activity score (NAS ≥ 4), and/or advanced fibrosis (F ≥ 2). Active disease is identified by a high cut-off of ≥0.67 and is ruled out by a low cut-off of ≤0.35 with an AUROC of 0.8 [52].

In patients with ALD using TE to stratify the stage of hepatic fibrosis, the meta-analysis demonstrated a cut-off of 9.0 kPa to identify significant fibrosis (F ≥ 2) with an AUROC of 0.86, 12.1 kPa to identify advanced fibrosis (F ≥ 3) with an AUROC of 0.9, and 18.6 kPa to identify cirrhosis (F4) with an AUROC of 0.91. The author also noted that liver stiffness was influenced by high AST and bilirubin concentrations [53]. In comparison with the serum NIT biomarkers FibroTest, APRI, PGA, and PGAA, the accuracy of TE is superior. A combination of TE and these biomarkers did not improve performance [54].

### 3.3. Magnetic Resonance Elastography (MRE)

Magnetic Resonance Elastograph (MRE) is a non-invasive MRI-based method that uses a low frequency propagating wave (60 Hz vibrations) with phase-contrast to measure liver stiffness. It processes every 6–10 mm of liver parenchyma [55]. In a cross-sectional prospective well-designed study of 117 biopsy-proven NAFLD patients who underwent MRE, the AUROC for detecting advanced fibrosis (F ≥ 3) was 0.924 at a cut-off of 3.63 kPa with 86% sensitivity, 91% specificity, 68% PPV, and 97% NPV. [56] Another analysis from a phase II trial of Selonsertib from 23 sites in the US and Canada demonstrated MRE cut-offs of 4.7 kPa and 5.5 kPa to detect advanced fibrosis (F ≥ 3) and cirrhosis (F4), respectively, with an AUROC of 0.8 [57]. In a recent multi-center study of patients with NAFLD, a cut-off of 4.39 kPa was shown to detect cirrhosis (F4) with an AUROC of 0.92 [58].

The accuracy of predicting hepatic fibrosis in NAFLD patients was compared between MRE and TE. In a study by Park et al., the AUROCs for predicting advanced fibrosis (F ≥ 3) (at a cut-off of 2.99 kPa) and cirrhosis (F4) (at a cut-off of 3.35 kPa) by MRE were 0.87 and 0.87, respectively, while those for predicting advanced fibrosis and cirrhosis by TE were 0.80 and 0.69 [59]. Another comparison study among Japanese NAFLD patients demonstrated high cut-offs of 4.8 kPa and 6.7 kPa for detecting advanced fibrosis (F ≥ 3) and cirrhosis (F4) with AUROCs of 0.89 and 0.97, respectively [60]. In a pooled analysis, MRE outperformed TE across different stages of hepatic fibrosis: The AUROCs of MRE vs. TE for stage 1 fibrosis were 0.87 vs. 0.82, those for stage 2 fibrosis were 0.92 vs. 0.87, those for advanced fibrosis (F ≥ 3) were 0.93 vs. 0.84, and those for cirrhosis (F4) were 0.94 vs. 0.84, which were all statistically significantly different [61]. Interestingly, the rates for determining a discordant fibrosis stage (compared to the biopsy-proven hepatic fibrosis stage) in TE (51.9% in the training cohort and 58.8% in the validation cohort) were higher than those in MRE (21% in the training cohort and 14.7% in the validation cohort), especially in obese patients. The discordance rates were significantly worse in patients with BMI >35 kg/m^2^ [62].

The accuracy of MRE was also compared with serum NIT biomarkers including the FIB4-index, AAR, NFS, BARD, and APRI score to predict advanced fibrosis (F ≥ 3). The performance of MRE is superior to that of serum NIT biomarkers given that the AUROC of MRE was 0.97 at a cut-off of 3.64 kPa, which was higher than AUROCs of FIB-4 (0.861), AAR (0.825), NFS (0.818), BARD (0.816), and APRI (0.807), respectively [63]. The major limitations of MRE include limited availability, technical complexity with requirement of special hardware, high cost, and contraindication in claustrophobic patients. There is currently no well-designed study to determine the optimal cut-off or accuracy of MRE to stratify different stages of hepatic fibrosis among patients with alcoholic liver disease. The summary of accuracy is shown in Table 3.

## 4. Staging Algorithm to Assess Hepatic Fibrosis

**Figure 1:**
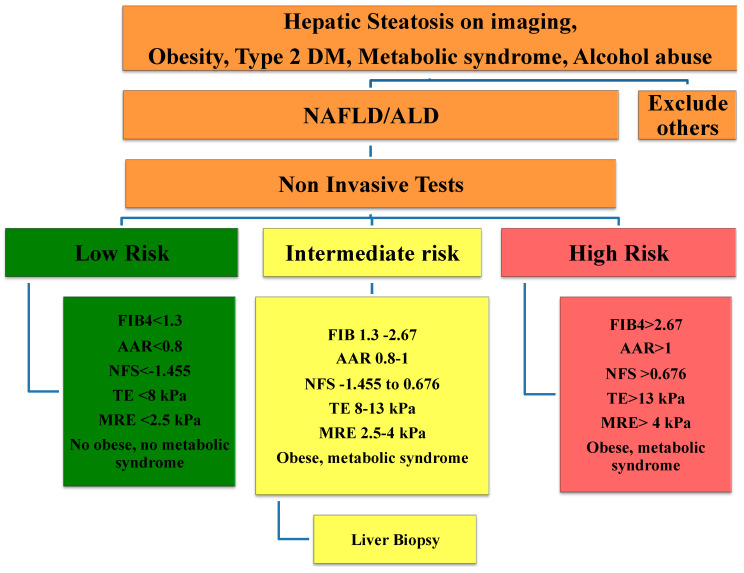
Algorithm to assess hepatic fibrosis. NFS = NAFLD fibrosis score, AAR = AST/ALT ratio, TE = transient Elastography, MRE = magnetic resonance elastography. Figure 1 is adapted from previously published articles.

Based on the evidence in the literature, we proposed an algorithm to stage or assess the severity of hepatic fibrosis. When patients are suspected to have fatty liver syndrome, including the presence of steatosis on imaging, or possess risk factors such as obesity, metabolic syndromes, and/ or a history of alcohol consumption, the first step is to exclude other possible etiologies, such as Wilson disease, viral hepatitis, and autoimmune liver disease. The next step is to stratify the risk of the patients utilizing non-invasive tests. If the patients do not have any risks factors and present non-invasive serum biomarkers below the low cut-offs, have a liver stiffness value measured by TE below 8 kPa, or present liver stiffness measured by MRE below 2.5 kPa, there is no need for further investigation. If the patient falls into a high-risk category, such as having non-invasive serum biomarkers above the high cut-offs or liver stiffness measured by TE above 13 kPa or that measured by MRE above 4 kPa or the presence of other risk factors, the patients are likely to have advanced fibrosis or cirrhosis. If the patient’s non-invasive serum biomarkers fall within the indeterminate zone, or if they present a liver stiffness measured by TE between 8 and 13 kPa or between 2.5–4 kPa when measured by MRE alongside risk factors such as obesity, metabolic syndrome, or a history of alcohol consumption, a liver biopsy is suggested to further assess the stage of hepatic fibrosis accurately (Figure 1).

## 5. Prediction of Mortality and Liver-Related Outcomes

The evidence in the literature suggests that histologic hepatic fibrosis assessed by liver biopsy can predict mortality and liver-related outcomes. Liver-related outcomes include hepatic decompensation (variceal bleeding, ascites, hepatic encephalopathy, hepato–renal syndrome, hepato–pulmonary syndrome, hepatic hydrothorax, etc.), liver failure, and hepatocellular carcinoma [5,6,7,8]. These predictions were also explored for non-invasive biomarkers and imaging tests.

### 5.1. Prediction with Serum-Based Biomarkers

A retrospective multi-center international study of 320 biopsy-proven NALD patients revealed that the NFS, APRI, FIB4-index, and BARD score were able to estimate liver-related events, mortality, and liver transplantation with a high hazard ratio (HR). Among those scores, the best predictor was NFS, as its HR values for liver-related events were 7.7 and 34.2, while the HR for mortality and liver transplantation was 4.2 and 9.8 among intermediate-risk and high-risk groups, respectively [64]. Another retrospective analysis from Sweden investigated the accuracy of non-invasive serum biomarkers to predict mortality and liver-related outcomes in 646 biopsy-proven NAFLD patients. The AUROCs of NFS (0.72) and the FIB-4 index (0.72) were better in predicting mortality than those of BARD (0.62) and APRI (0.52). Similarly, better AUROCs of NFS (0.72) and the FIB-4 index (0.72) were found compared to those of BARD (0.62) and APRI (0.69) to predict severe liver-related outcomes, including decompensated liver disease, liver failure, and hepatocellular carcinoma [65]. The accuracy of all-cause and liver-related mortality or liver transplantation, as well as liver-related outcomes including cirrhosis, hepatic decompensation, and hepatocellular carcinoma, were compared between NAFLD patients with and without diabetes mellitus using APRI and FIB-4. Compared to the NAFLD patients without diabetes mellitus, APRI and FIB-4 in patients with diabetes mellitus were less accurate in predicting overall mortality/liver transplantation, liver-related outcomes, and liver-related mortality [66]. The enhanced liver fibrosis score (ELF) is also useful for predicting liver-related outcomes in patients with NASH and decompensated cirrhosis based on data extrapolated from a phase 2 randomized controlled trial of Belapectin (NCT02462967). Liver related outcomes in this study included the incidence or worsening of gastroesophageal varices, variceal hemorrhaging, the occurrence of new ascites, hepatic encephalopathy, an increase in the Child–Turcotte–Pugh (CTP) score of ≥2 points from baseline or a rise in the MELD score to >15. Patients with ELF ≥11.3 were more likely to develop liver-related events with a cox proportional hazard ratio (HR) of 4.81 compared to patients with an ELF <9.8. The AUROC of baseline ELF was 0.67, and that after increasing ELF overtime was 0.68 for predicting liver-related outcomes [67]. Similarly, the AUROCs for the serum biomarkers of FibroTest, FIB4, APRI, and Forns index were 0.79, 0.65, 0.60, and 0.40, respectively, for predicting non-liver-related mortality and 0.69, 0.64, 0.57, and 0.43, respectively, for predicting overall mortality in patients with ALD [20].

### 5.2. Predictions with Imaging Tests

The liver stiffness measured by transient elastography provided similar accuracy to the portal pressure measurement (Hepatic Venous Portal Gradient, HVPG), which is the gold standard to predict portal hypertension at a cut-off of 21.1 kPa, as shown in a prospective study by Robic et al. [68]. Patients higher in transient elastography based liver stiffness, especially F4 defined by TE, were found to have lower survival in another prospective study of 360 patients with NAFLD [69]. In a retrospective analysis of NAFLD patients, high baseline TE-based liver stiffness and a change in liver stiffness within 6 months were associated with hepatic decompensation, hepatocellular carcinoma, liver-related mortality, and overall mortality (HRs of 1.56, 1.72, 1.96, 1.73, respectively) [70].

Studies have shown that liver stiffness measured by MRE can accurately diagnose portal hypertension defined by HVPG in chronic liver disease patients [71,72]. The baseline liver stiffness value measured by MRE also predicts hepatic decompensation. Patients with compensated liver disease with baseline liver stiffness value ≥5.8 kPa had an HR of 4.96 for hepatic decompensation when compared to those with a low baseline value [73]. A recent multi-center NAFLD cohort study demonstrated that the baseline MRE based liver stiffness can predict hepatic decompensation, including ascites, hepatic encephalopathy, esophageal variceal bleeding, and mortality. The odds of hepatic decompensation increased 3.28-fold with an increase of 1 kPa in liver stiffness over time. The cut-off for the liver stiffness value to predict hepatic decompensation was 6.48 kPa, with an AUROC of 0.707, 66.7% sensitivity, 80.8% specificity, and 73.7% accuracy. This study also defined the median cut-offs for individual decompensation events: 7.1 kPa for the occurrence of ascites, 8.85 kPa for hepatic encephalopathy, and 10.1 kPa for esophageal variceal bleeding and mortality [58].

## 6. Non-Invasive Tests to Monitor Treatment Response

Finally, the role of non-invasive tests (both serum biomarkers and imaging tests) in the monitoring of treatment response is integral in phase 2 clinical trials. A reduction of 10 U/L in alanine aminotransferase (ALT) was shown to be associated with histologic improvements and NASH resolution [74]. Moreover, a reduction of ≥17 IU/L in ALT was able to predict histologic response with an AUROC of 0.83 [75]. The MRI-proton density fat fraction (MRI-PDFF) non-invasively measures the percentage of fat in the liver. An absolute reduction of ≥5% in the MRI-PDFF value was found to be associated with regression in steatosis with 90% specificity and 58% sensitivity [76]. A relative reduction of ≥30% in the MRI-PDFF value was associated with improvement in the NAFLD activity score without the worsening of fibrosis [77]. When liver stiffness measured by MRE was evaluated for treatment response among 54 NAFLD patients, a reduction in liver stiffness of at least 2.3% was associated with fibrosis improvement. Any percentage of relative reduction (≥0%) in liver stiffness measured by MRE can predict fibrosis improvement with 67% sensitivity, 64% specificity, 48% PPV, 79% NPV, and AUROC of 0.79. Similarly, fibrosis progression can be also detected by MRE-liver stiffness [57]. Other complex non-invasive serum biomarkers, such as ELF, Pro-C3, and liver stiffness measured by TE, were proposed for use in the monitoring of treatment response [78]. When the treatment response assessed by histology was compared with percent change in NIT tests, AUROC of MRE (0.617) was superior compared to that of MRI-PDFF (0.515), NFS (0.561), FIB-4 (0.585), TE (0.578), and ELF score (0.581) [57]. Further investigations into the non-invasive tests for monitoring treatment response are warranted. 

## 7. Conclusions

In summary, the staging of hepatic fibrosis in fatty liver syndrome is essential. The utilization of non-invasive tests to assess the staging of liver disease has become an acceptable alternative to liver biopsy. Among the simple non-invasive biomarkers, the FIB4-index and NFS provide the best accuracy in identifying advanced fibrosis or cirrhosis. New complex serum biomarkers are presently evolving with promising accuracy. Moreover, the performance of MRE is superior to that of TE and SWE in assessing hepatic fibrosis. The roles of non-invasive tests are emerging but are not limited to risk stratification, the prediction of disease outcomes, and the monitoring of treatment response. 

## Figures and Tables

**Table 1:** Accuracy of simple biomarkers used to identify advanced fibrosis in both non-alcoholic fatty liver disease (NAFLD) and alcoholic fatty liver disease (ALD).

Simple Biomarkers	Cut-Off	AUROC in NAFLD	AUROC in ALD
NAFLD fibrosis score (NFS)	0.676	0.81–0.84	NA
AST to Platelet Ratio Index (APRI)	1.5–2.0	0.67–0.8	0.67–0.85
FIB4-Index	2.67	0.80–0.86	0.8–0.85
BARD score	2–4	0.73–0.81	NA
AST/ALT Ratio (AAR)	1	0.74–0.83	NA
Forns Index	4.1	NA	0.38–0.89

AUROC = area under the receiver operating characteristics curve.

**Table 2:** Accuracy of complex biomarkers to identify advanced fibrosis in both non-alcoholic fatty liver disease (NAFLD) and alcoholic fatty liver disease (ALD).

Complex Biomarkers	Cut-Off	AUROC in NAFLD	AUROC in ALD
FibroTest/FibroSure	0.7	0.81	0.83–0.88
Fibrospect	17	0.85–0.86	NA
Enhanced Liver Fibrosis Panel	0.357–0.462	0.87–0.9	0.92
Pro-C3 based predictive fibrosis score	6.328	0.86–0.87	NA
NIS4	0.6137	0.826	NA
PGAA index	10	NA	0.87

AUROC = area under the receiver operating characteristics curve.

**Table 3:** Accuracy of imaging tests to identify advanced fibrosis in both non-alcoholic fatty liver disease (NAFLD) and alcoholic liver disease (ALD).

Imaging	Cut-Off	AUROC in NAFLD	AUROC in ALD
pSWE	1.77 m/s	0.91–0.97	0.87
2D SWE	10.2 kPa	0.89	0.92
TE	8–12.1 kPa	0.8–0.87	0.9
MRE	3.64–4.7kPa	0.8–0.97	No well-designed study

AUROC = area under the receiver operating characteristics curve, pSWE = point shear wave elastography, 2D SWE = two-dimensional shear wave elastography, TE = transient elastography, MRE = magnetic resonance elastography.

## Abbreviations

AAR	AST/ALT Ratio
ALD	Alcoholic Liver Disease
APRI	AST to Platelet Ratio Index
AUROC	Area under the Receiver Operating Characteristic Curve
ELF	Enhanced Liver Fibrosis Panel
MRE	Magnetic Resonance Elastography
NAFLD	Non-alcoholic Fatty Liver Disease
NASH	Non-alcoholic steatohepatitis
NFS	Non-alcoholic Fatty Liver Disease Fibrosis Score
SWE	Shear wave Elastography
TE	Transient Elastography
